# Multi-target regulatory mechanism of Yang Xin Tang − a traditional Chinese medicine against dementia

**DOI:** 10.1186/s13020-023-00813-w

**Published:** 2023-08-16

**Authors:** Tung Yan Lo, Anthony Siu Lung Chan, Suet Ting Cheung, Lisa Ying Yung, Manton Man Hon Leung, Yung Hou Wong

**Affiliations:** 1 Division of Life Science and the Biotechnology Research Institute, Hong Kong University of Science and Technology, Hong Kong, China; 2 State Key Laboratory of Molecular Neuroscience, Molecular Neuroscience Center, Hong Kong University of Science and Technology, Hong Kong, China; 3 Center for Aging Science, Hong Kong University of Science and Technology, Hong Kong, China; 4 Hong Kong Center for Neurodegenerative Diseases, Units 1501-1502, 17 Science Park West Avenue, Hong Kong Science Park, Shatin, New Territories, Hong Kong, China; 5 Present Address: Sir William Dunn School of Pathology, University of Oxford, Oxford, UK

**Keywords:** Alzheimer’s disease, β amyloid, Chinese medicine, Neural progenitor cell, Neurodegenerative disease, Neuroprotection, Yang Xin Tang

## Abstract

**Background:**

Yang Xin Tang (YXT) is a traditional Chinese herbal preparation which has been reported to improve cognitive function and memory in patients with dementia. As the underlying mechanism of action of YXT has not been elucidated, we examined the effects of YXT and its major herbal components in regulating gene transcription and molecular targets related to Alzheimer’s disease (AD).

**Methods:**

Aqueous and ethanol extracts of YXT and selected herbal components were prepared and validated by standard methods. A series of biochemical and cellular assays were employed to assess the ability of the herbal extracts to inhibit acetylcholinesterase, reduce β-amyloid aggregation, stimulate the differentiation of neural progenitor cells, suppress cyclooxygenase, and protect neurons against β-amyloid or N-methyl-D-aspartate-induced cytotoxicity. The effects of YXT on multiple molecular targets were further corroborated by a panel of nine reporter gene assays.

**Results:**

Extracts of YXT and two of its constituent herbs, *Poria cocos* and *Poria Sclerotium pararadicis,* significantly inhibited β-amyloid aggregation and β-amyloid-induced cytotoxicity. A protective effect of the YXT extract was similarly observed against N-methyl-D-aspartate-induced cytotoxicity in primary neurons, and this activity was shared by extracts of *Radix Astragali* and *Rhizoma Chuanxiong*. Although the YXT extract was ineffective, extracts of *Poria cocos*, *Poria Sclerotium pararadicis* and *Radix Polygalae* inhibited acetylcholine esterase, with the latter also capable of upregulating choline acetyltransferase. YXT and its components significantly inhibited the activities of the pro-inflammatory cyclooxygenases. Additionally, extracts of YXT and several of its constituent herbs significantly stimulated the phosphorylation of extracellular signal-regulated kinases and cAMP-responsive element binding protein, two molecular targets involved in learning and memory, as well as in the regulation of neurogenesis.

**Conclusions:**

Several constituents of YXT possess multiple regulatory effects on known therapeutic targets of AD that range from β-amyloid to acetylcholinesterase. The demonstrated neuroprotective and neurogenic actions of YXT lend credence to its use as an alternative medicine for treating AD.

**Supplementary Information:**

The online version contains supplementary material available at 10.1186/s13020-023-00813-w.

## Introduction

Alzheimer’s disease (AD) is a common age-associated syndrome which contributes to 70% of dementia cases worldwide, and the need for AD therapeutics will increase tremendously in the foreseeable future. Current medications, including acetylcholinesterase (AChE) inhibitors and N-methyl D-aspartate (NMDA) antagonists [1, 2], can impede disease progression and are primarily effective in the early stages of AD. However, they are unable to cure or reverse the disease. Due to the complex pathophysiology of AD, the traditional approach of drug development for identifying a drug that acts on a specific molecular target has proven unproductive [3]. Hence, there is an urgent need to explore alternative approaches to relieve the medical and societal burdens associated with AD. In this regard, traditional Chinese medicine (TCM) that have a long history of clinical use may hold great promise in delaying or halting the progression of the patient’s symptoms (e.g., loss of memory and impaired cognition).

TCM is well-known for its multi-target properties as each composite formula contains numerous chemical constituents. Modern pharmacological studies have evaluated the effects of TCM-derived molecules in treating dementia [4] and unveiled compounds that possess neuropharmacological activities with beneficial effects against neurodegenerative diseases [5]. For example, osthole from *Cnidium monnier L. Cusson* can reduce the β-amyloid (Aβ) load by up-regulating miR-107 [6], while emodin from *Rheum officinale* Baill is known to suppress Aβ deposition and tau phosphorylation [7]. Several TCM formula or naturally derived bioactives have indeed been tested in clinical trials, including EGb761 (extracted from *Ginkgo biloba*) [8] and sodium oligomannate (isolated from *Ecklonia kurome*) [9] for AD, as well as Pingchang granule for Parkinson’s disease [10]. As modernization and clinical application of TCM is gaining momentum, re-engineering of TCM formula may yield new anti-AD therapeutic options.

Yang Xin Tang (YXT) is a complex TCM formula comprised of 13 Chinese medicinal herbs, including *Poria cocos* (PA; Fuling), *Poriae Sclerotium pararadicis* (PS; Fushen), *Radix Astragali* (RA; Huangqi), *Rhizoma Chuanxiong* (RC; Chuanxiong), *Radix Angelica Sinensis* (RS; Danggui), *Radix et Rhizoma Glycyrrhizae* (RG; Gancao), *Radix Polygalae* (RP; Yuanzhi), *Rhizoma Pinelliae Preparatum* (PP; Banxia), *Fructus Schisandrae* (FS; Wuweizi), *Cortex Cinnamomi* (CC; Rougui), *Semen Platycladi* (SP; Baiziren), *Radix Ginseng* (GS; Renshen) and *Semen Ziziphi spinosae* (SZ; Suanzaoren). Many of these herbs have been shown to improve cognition and are frequently included in concoctions for treating senile dementia [11]. YXT was first recorded in *Effective Recipes from Renzhai House* (Renzhai Zhi Zhi Fang Lun) with multiple versions being described to date. The *Complete Library in the Four Branches of Literature* (Si Ku Quan Shu) contained the most authoritative YXT prescription in known literature, which is the version adopted in this study. Reports on the elucidation of YXT prescription are scarce. In the treatment of blood circulation disorder and its associated mental/intellectual dysregulation, RA and GS act as the monarch (jun) herbs due to their powerful action to tonify the Qi of spleen, while PA, PS, and RS are believed to serve as minister (chen) herbs to strengthen the therapeutic effects [12]. As PA and PS are well-known for their sedative role, they are also used to calm patients, whereas RS is used to promote circulation. Likewise, assistant (zuo) herbs including CC, FS, PP, RC, RP, SP, and SZ cooperatively calm the spirit of patients as typical tranquilizers [12]. As for guide/courier (shi) herbs, RG is used for harmonizing the whole formula as it can guide the therapeutic actions of different herbal medicines to their respective target meridians.

Contemporary TCM practitioners generally regard AD as a result of imbalance in heart yin-yang and deficiency of kidney essence [13]. Hence, herbal treatment of AD typically aims to restore the yin-yang balance in the kidney and heart, as well as promoting the Qi and blood circulation in the brain. Not surprisingly, most of the components of YXT are categorized as Qi- and blood-invigorating herbs, several of which apparently possess multiple anti-AD activities. For instance, RA has an outstanding neuroprotective effect against toxin-induced neuronal cell death and is known to improve cognition in memory-impaired animals [14]. Similarly, RP attenuates scopolamine-induced memory impairment [15] and promotes the proliferation and differentiation of neural stem cells [16]. These observations highlighted the potential of YXT being a treatment option for AD. In order to elucidate the mechanistic basis of YXT, we examined the anti-AD properties of YXT and attempted to identify the relevant molecular targets. Since YXT is likely to affect multiple targets and signaling pathways implicated in AD pathogenesis, we investigated whether the formula or its herbal components can modulate gene transcription and/or the function of selected molecular targets of AD. Accordingly, the potential beneficial actions of YXT on AD-related targets such as enzymes, kinases, and signaling molecules were examined by biochemical and cellular assays. Likewise, regulatory elements associated with neurogenesis, loss of neurons, or the formation of Aβ plaques and neurofibrillary tangles were assessed by luciferase reporter gene assays. Our study revealed that YXT and several of its herbal components apparently possess neuropharmacological properties that may be beneficial against AD.

## Materials and methods

### Reagents and materials

Herbal materials were purchased from Lee Hoong Kee Limited (Hong Kong) and Eu Yan Sang (Hong Kong). Antibodies including anti-neurofilament-L, anti-neurofilament-M, anti-neurofilament-H, anti-GFAP, anti-βIII-tubulin, anti-β-actin, anti-ChAT, anti-phospho-ERK, anti-ERK, anti-phospho-CREB, and anti-CREB were obtained from Cell Signaling Technology (MA, USA). Chemical markers for high-performance liquid chromatography (HPLC) reference were purchased from Cheungdu Biopurify Phytochemicals Ltd. (China), Fujifilm Wako Chemical Corporation (Japan), and Aobious Inc. (MA, USA). ReNcell culture materials including ReNcell^™^ VM neural progenitor cell line (Cat# SCC008) and ReNcell NSC maintenance medium (Cat#SCM005) were purchased from EMD Millipore. Other cell culture materials were purchased from Thermo Fisher Scientific (OH, USA) and Millipore Sigma (MA, USA). SensoLyte^®^Thioflavin T Aβ42 Aggregation kit (Cat# AS-72214) were purchased from AnaSpec (CA, USA). COX-1 and COX-2 inhibitor screening kits were from BioVision Inc. (CA, USA).

### Extraction and authentication of herbal materials

The YXT formula utilized in this study represents one of the original recipes which was recorded in the *Complete Library in the Four Branches of Literature*. The ratio of each herb was calculated by converting the Chinese unit of weight (catty, tael, mace, candareen) to an international unit of weight (gram), and the individual herbal components were weighed and mixed before extraction. For ethanol extraction of herbs, herbal materials (100 g) were macerated in 600 mL of 70% ethanol for 15–20 min, followed by boiling with reflux for 1 h. The residues were subjected to a re-extraction under identical conditions. The extracts were pooled and filtered, then transferred to a rotary evaporator and concentrated under reduced pressure at 60 °C. The extracts were then lyophilized to remove the water content for long-term storage. Water extraction of herbal materials was conducted in a similar manner, except the solvent was replaced with distilled water. For the YXT formula (100 g), the following herbs (with their origin indicated in parenthesis) were weighed and mixed before extraction: 14.5 g each of PA (Yunnan), PS (Hubei), RA (Shanxi), RC (Sichuan), PP (Sichuan), and RS (Gansu); 11.5 g of RG (Inner Mongolia); 0.3 g each of CC (Guangxi), FS (Ningxia), GS (Jilin), RP (Shaanxi), SP (Hubei), and SZ (Hubei). All herbal materials utilized in this study are daodi herb to ensure the best pharmacological properties of herbal extracts, and these materials were authenticated by HPLC against chemical markers listed in Additional file 1: Table S1.

### Cell culture

Human embryonic kidney 293 (HEK293; CRL-1573), SH-SY5Y human neuroblastoma (CRL-2266), U-87 MG human glioblastoma (HTB-14), and PC12 rat pheochromocytoma (CRL-1721) cells were obtained from American Type Culture Collection (VA, USA). Cells were grown on 100 mm culture plates with Dulbecco’s modified Eagle’s medium containing 100 units/mL penicillin and 100 μg/mL streptomycin, and supplemented with 10% fetal bovine serum or 5% fetal bovine serum and 10% horse serum according to the manufacturer’s recommendation. Cells were maintained in a humidified CO_2_ (5%) incubator at 37 °C. The human ReNcell^™^ VM neural progenitor cell line was grown in ReNcell NSC maintenance medium supplemented with 20 ng/mL basic fibroblast growth factor and 20 ng/mL epidermal growth factor until 70% confluency. Growth factors were then withdrawn from the culture medium for 7 days to differentiate ReNcell into a mixed culture (ReNcell-derived immature neurons; RDIN) composed of immature neurons (80%), astrocytes (15%), and oligodendrocytes (5%) according to the manufacturer’s protocol.

### Primary cultures of cortical neurons

Primary mouse cortical neuronal cultures were obtained from E16 ICR mouse embryos (Laboratory Animal Facility, Hong Kong University of Science and Technology). Briefly, cerebral cortices were removed from E16 ICR mouse embryos and mechanically dissociated in PBS with glucose (18 mM). Neurons were then seeded onto poly-L-lysine (1 mg/mL) coated 96-well plates at 5 × 10^4^ cells/well in B27 Plus Neuronal culture system (Thermo Fisher Scientific), supplemented with 2 mM glutamine, 5 μM mercaptoethanol, 100 U/mL penicillin and 100 μg/mL streptomycin. The cultured neurons were maintained in a humidified incubator at 37 ℃ with 5% CO_2_ for 7 days prior to use.

### DNA constructs and transfection 

Luciferase reporter constructs including pAP-1-Luc, pCRE-Luc, pGAS-TA-Luc, p-ISRE-TA-Luc, pLILRE-Luc, pNFκB-Luc, p53-Luc, pSTAT3-TA-Luc, and pTARE-Luc were purchased from Agilent Technologies (CA, USA) and Clontech Laboratories, Inc (Japan). HEK293 cells in 100 mm plates at 70–80% confluency were transfected with different reporter plasmids by Lipofectamine 3000 (Thermo Fisher Scientific). Stable transfectants were selected with 75 μg/mL Zeocin for pcDNA3.1-zeo or 400 μg/mL G418 for pcDNA-neo.

### Luciferase reporter assay 

HEK293 cells seeded in 12-well plates were serum starved overnight prior to herbal treatment for 24 h. All herbal extracts (both aqueous and ethanol) were applied at 100 μg/mL, except RP was used at 10 μg/mL. Cells were incubated in luciferase cell lysis buffer for 2 h (Promega, WI, USA). The luciferase assay was performed as described previously [17] and the luciferase activities were expressed in relative fluorescence units (RFU).

### SDS-PAGE and western blot analysis

Cells were treated with herbal extracts for different durations as indicated in the figures, and cell lysates were prepared as described previously [18]. Samples were resolved on 4–12% NuPAGE Bis–Tris gels (ThermoFisher Scientific) and then transferred to nitrocellulose membrane. Resolved proteins were detected by specific primary antibodies. Membranes were washed twice with Tris-buffer saline containing 0.1% Tween 20, followed by an incubation with horseradish peroxidase-linked secondary antisera (Cytiva, WA, USA). Immunoblots were visualized by Western Bright enhanced chemiluminescence kit (Advansta, CA, USA) according to the manufacturer’s protocol.

### Aβ aggregation assay

Aggregation of Aβ was monitored by using the SensoLyte^®^Thioflavin T Aβ42 Aggregation kit. A mixture of Aβ42 peptide (5 µM) and test sample was added to the 2 mM Thioflavin T (ThT) solution in each well of a 96-well plate, and the aggregation of Aβ was monitored over 100 min. Fibrillation was measured by the fluorescence intensity at 37 °C with Ex/Em = 440 nm/484 nm using a SpectraMax Gemini XS microplate reader (Molecular devices, CA, USA). The reading from a blank control was used as the background fluorescence, and all readings were expressed in RFU.

### Cell viability assay

The water-soluble tetrazolium salts-1 (WST-1) cell viability assay was used to evaluate the viability of NMDA- or Aβ-treated primary neuronal cultures. Herbal treatments were applied to the cultured primary neurons 2 h prior to the addition of 5 μM Aβ42 oligomers (AnaSpec) or 100 μM NMDA (Calbiochem, CA, USA). Following incubation at 37 °C for 48 h, WST-1 solution (10 μL/well; Millipore Sigma) was added to the 96-well plate and incubated for 1 h. The absorbance at 450 nm was measured with a microplate reader to determine the formation of formazan dye produced by viable cells, with 650 nm as the reference wavelength.

### Determination of AChE activity 

AChE activity was determined by the Ellman assay [19]. Briefly, lysates of PC12 cells were incubated with the substrate-containing reaction buffer in the absence or presence of herbal extracts at 37 °C for 30 min, and the absorbance at 405 nm was then measured.

### Measurement of COX-1 and COX-2 enzymatic activity

The enzymatic activities of COX were measured using COX-1 and COX-2 inhibitor screening kits. Assays were performed following manufacturer’s protocol. In brief, ovine COX-1 or human recombinant COX-2 were reconstituted in assay buffer containing COX enzymes (COX-1 or -2), COX cofactor, arachidonic acid in NaOH, and a fluorescence probe. After a 10 min incubation at room temperature, the COX-activity was measured by the fluorescence intensity with Ex/Em = 535/587 nm using SpectraMax Gemini XS microplate reader. All readings were expressed in RFU. COX-1 specific inhibitor (SC560) and COX-2 specific inhibitor (celecoxib) served as controls.

### Quantification and statistical analysis

Immunoreactive bands were quantified using ImageJ analysis software (National Institutes of Health, MD, USA). Experimental data were statistically analyzed by GraphPad Prism version 7.0 for PC (GraphPad Software, CA, USA). The data were analyzed using one-way analysis of variance (ANOVA) test, then further compared to basal or control group by using Dunnett’s test. P-values of less than 0.05 were defined as statistically significant. Independent experiments were typically performed in triplicates with the number of repeats (n values) indicted in the corresponding figure legends.

## Results

### Effects of YXT and selected herbal extracts on cell viability

Four major herbs of YXT including PA, PS, RA and RC were selected for analysis as each of them constitutes more than 15% of the whole formula by weight and they apparently possess neuropharmacological properties [14, 20, 21]. RP, a minor herb of YXT, was also selected since it is frequently included in TCM prescriptions for senile dementia [11]. Aqueous and ethanol extracts of YXT and its major/selected herbal components were prepared by standard methods and validated by HPLC determination of known chemical markers (Additional file 1: Table S1) as described under Materials and Methods. A WST cell viability assay was then performed to establish the maximal tolerated dose with no observable cytotoxic effect on human SH-SY5Y neuroblastoma, human U-87 MG glioblastoma and RDIN cultures, as well as HEK293 cells. Except for RP, aqueous and ethanol extracts of YXT and its selected components did not produce significant cytotoxic effects at 100 μg/mL on the cell lines tested (Additional file 1: Table S2). Thereupon, extracts were used at 100 μg/mL (except RP was used at 10 μg/mL) for subsequent cell-based assays; in vitro assays that utilized cell lysates were not subjected to this limitation.

### YXT and its herbal components inhibit Aβ aggregation and cytotoxicity

**Fig. 1:**
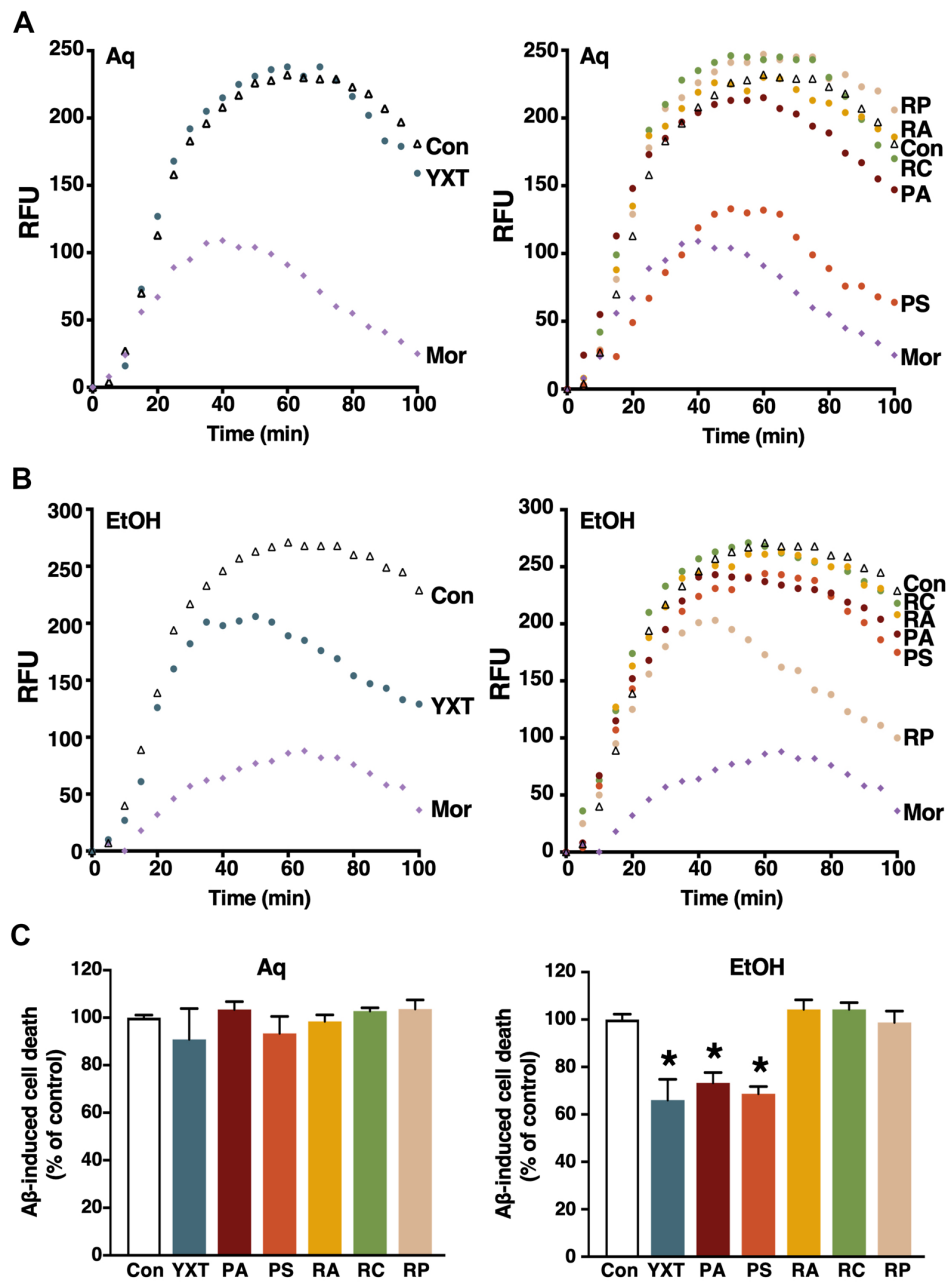
Effects of YXT and its selected herbal components on Aβ aggregation and cytotoxicity. **A** ThT assay was used to measure the aggregation of 5 µM Aβ42 in the absence (Con) or presence of (**A**) aqueous (Aq) or (**B**) ethanol (EtOH) extracts of YXT (left panel) and selected YXT herbal components (right panel). Data is illustrated in blue (YXT), dark red (PA), orange (PS), yellow (RA), green (RC), and beige (RP). A WST assay was used to assess Aβ42-induced cytotoxicity in primary cortical neurons cultured in the absence (Con) or presence of aqueous (left) or ethanol (right) extracts of YXT and its selected herbal components (**C**). Treatment was performed with extracts of RP at 10 μg/mL, and 100 μg/ mL for all the remaining herbs. 100% Aβ-induced cell death was defined as the loss of viable cells upon treatment with 5 µM Aβ42 for 48 h. *P < 0.05 (ANOVA); n = 6

Deposition of Aβ plaques is a hallmark of AD pathology. Aβ peptides are classified into two major isoforms composed of 40 or 42 amino acids (Aβ40 and Aβ42), with Aβ42 being the more toxic form which plays an essential role in the pathogenesis of AD. We investigated whether YXT and its herbal components could inhibit the Aβ42 aggregation by a ThT fluorescence assay. Morin (Mor; 100 μM) served as a positive control in this assay, as it has been shown to inhibit early stages of Aβ42 aggregation [22]. The aggregation level of Aβ42 was monitored over 100 min, during which the peak response occurred around 40–60 min. Inhibition of Aβ42 aggregation was illustrated by a sustained reduction in fluorescence signal by 100 min of treatment. The aqueous extract of YXT did not inhibit Aβ42 aggregation (Fig. 1A, left), yet, the aqueous extracts of PS clearly reduced Aβ42 aggregation by 65% (Fig. 1A, right). The aqueous extracts of PA, RA, RC, and RP did not affect Aβ42 aggregation. In contrast, the ethanol extract of YXT inhibited the Aβ42 aggregation by 43% (Fig. 1B, left). The ethanol extracts of PA, PS, RA and RC had no effect, while that of RP suppressed Aβ42 aggregation by 56% (Fig. 1B, right).

The ability of ethanol extracts of YXT and RP to suppress Aβ aggregation suggested that they may exert protective actions on Aβ42-induced neuronal toxicity. Primary neuronal cultures were incubated with or without herbal extracts for 2 h prior to treatment with 5 μM Aβ42 for 48 h, cells were then subjected to WST-1 assays. In the absence of herbal extracts, Aβ42 treatment reduced the viability of primary neuronal culture by ~ 50%. None of the aqueous extracts inhibited the Aβ42-induced toxicity (Fig. 1C, left). In contrast, ethanol extracts of YXT, PA, and PS significantly reduced Aβ42-induced cell death (Fig. 1C, right). Surprisingly, the ethanol extract of RP did not protect the neuronal culture from Aβ-induced cytotoxicity despite its ability to inhibit the aggregation of Aβ42.

### YXT and its herbal components inhibit AChE activity and stimulate ChAT expression

**Fig. 2:**
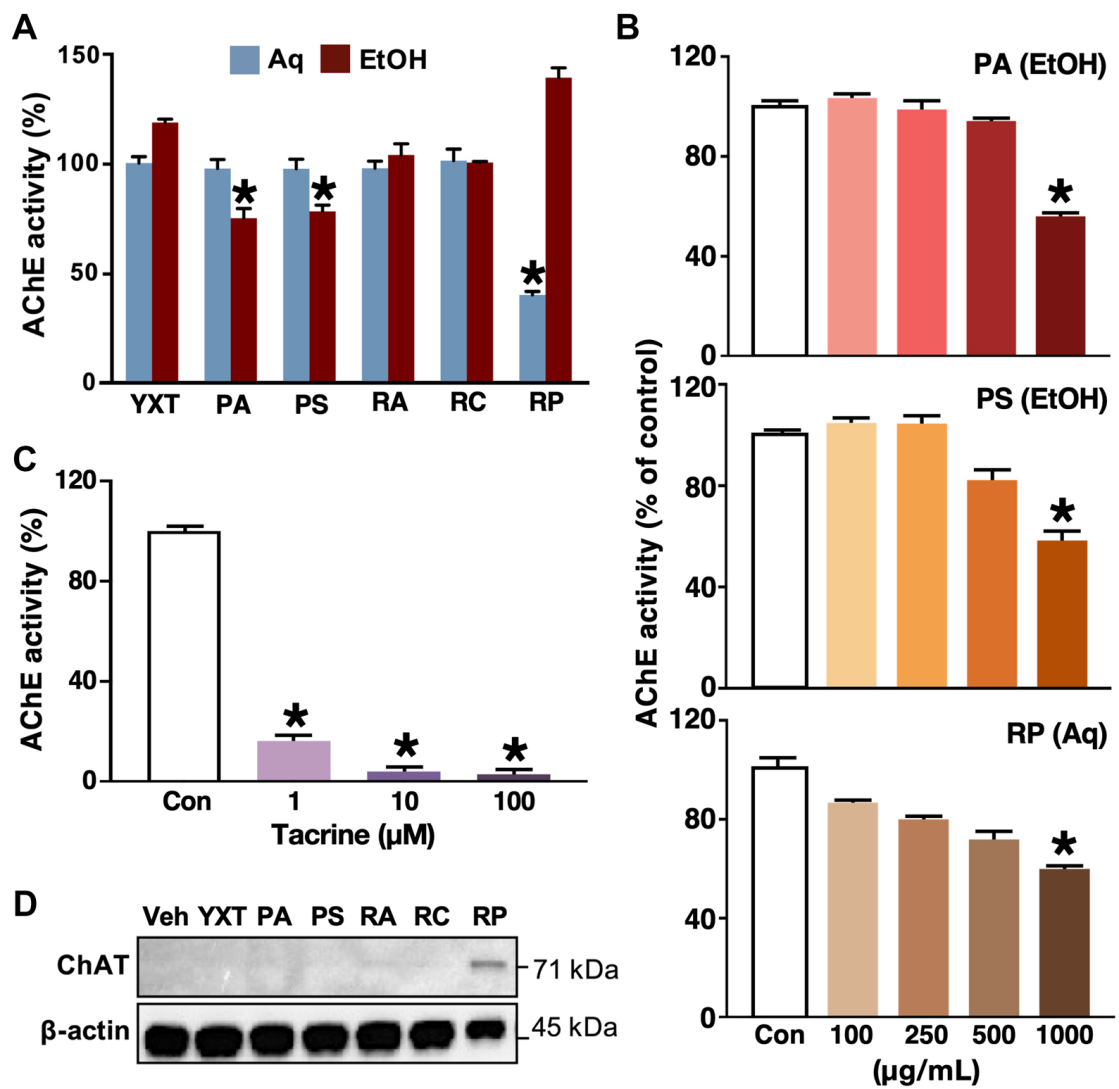
Effects of YXT and its selected herbal components on AChE activity and ChAT expression. **A** PC12 cell lysates were treated with extracts of YXT and its components at 1 mg/mL for 30 min, followed by an Ellman assay to determine the AChE activity (n = 6). **B** PC12 cell lysates were treated with ethanol extracts (EtOH) of PA, PS, and aqueous extract (Aq) of RP at various concentrations (100 µg/mL to 1 mg/mL) prior to the Ellman assay (n = 6). **C** Tacrine was used as a positive control for the Ellman assay, and the AChE activity measured in its absence was defined as 100% (Con). **D** RDIN cells were treated with the indicated herbal extracts for 7 days and analyzed by Western blotting to determine the amount of ChAT with β-actin as a loading control. The results were compared with basal group (Veh) in the absence of herbal treatment. *P < 0.05 (ANOVA); n = 6

Rectifying cholinergic system dysfunction is a mainstay therapeutic strategy for AD. Hence, we examined the effects of YXT and its herbal components on two key enzymes, AChE and choline acetyltransferase (ChAT). AChE hydrolyzes acetylcholine, and its inhibition enhances cholinergic neurotransmission. Lysates of PC12 cells (pheochromocytomas of neural crest origin) are commonly used to examine the enzymatic activity of AChE because they express the enzyme in abundance [23]. In the Ellman assay for determining AChE activity, the aqueous extract of RP and the ethanol extracts of PA and PS significantly suppressed the AChE activity (Fig. 2A). Dose-dependent inhibitory effects of these herbal extracts on AChE were discernable, albeit over a narrow concentration range of the herbal extracts (100–1000 μg/mL; Fig. 2B). The magnitudes of inhibition by these extracts were, however, lower than that of the positive control (Tacrine; Fig. 2C).

ChAT is a transferase that participates in the synthesis of acetylcholine. We determined the effects of YXT and its herbal components on the expression of ChAT in RDIN cells by Western blotting. RDIN is a mixed culture, typically composed of 85% immature neurons, 10% astrocytes, and 5% oligodendrocytes. It is derived from human neural progenitor cells with unique multipotent features for examining neuronal properties and neural lineage differentiation [24]. The ethanol extract of RP significantly induced ChAT expression after a 7 day treatment, while all other extracts tested did not show any induction of ChAT (Fig. 2D). Since expression of ChAT serves as a marker of mature cholinergic neuron, this implied that the number of mature neurons may increase upon treatment with RP. Together with the results on AChE inhibition, RP appeared to have a dual role in relieving the dysfunction of cholinergic system.

### YXT and its herbal components promote the differentiation of immature neurons to mature neurons 

**Fig. 3:**
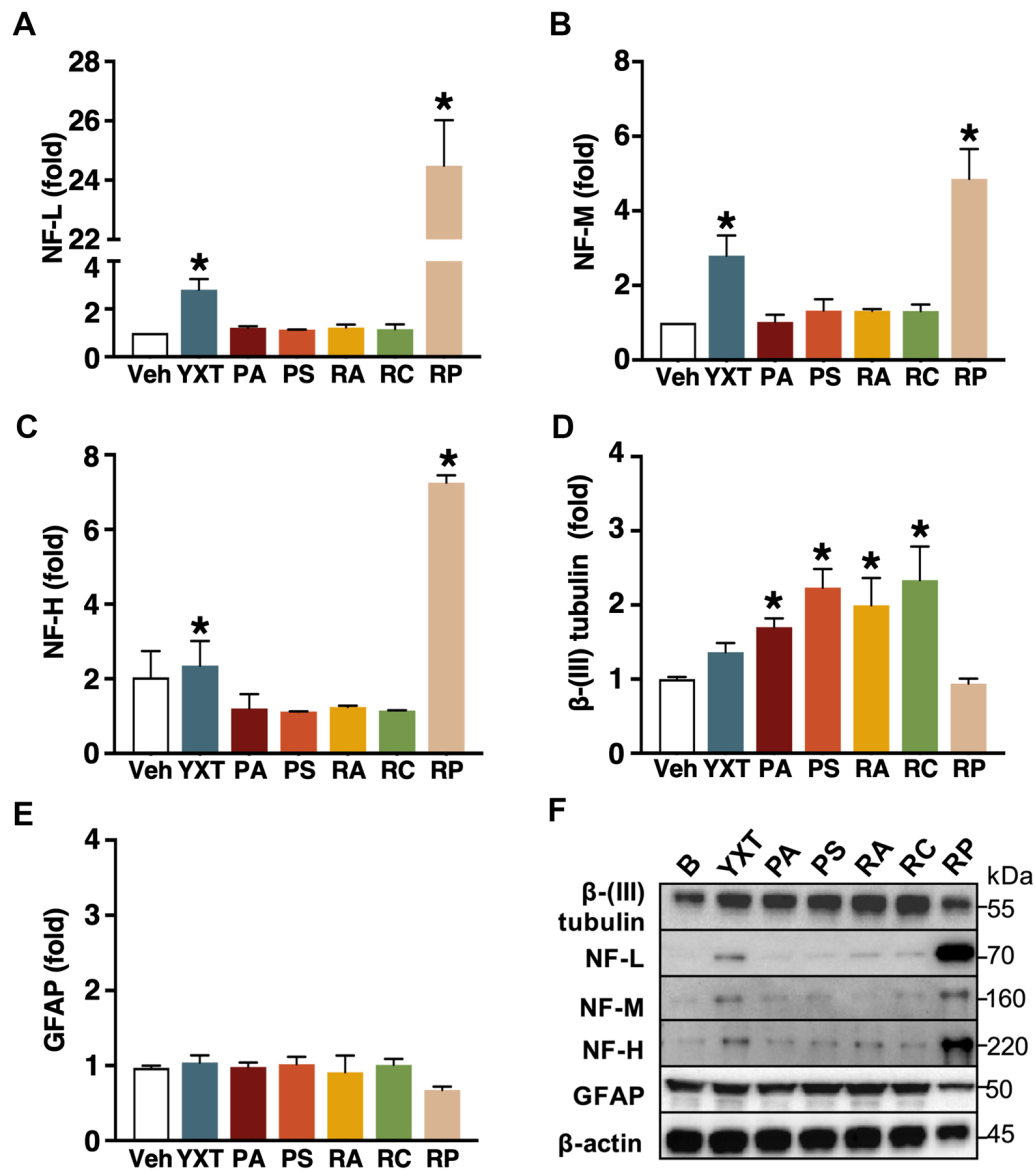
YXT and one of its herbal components promote the expression of neurofilaments. RDIN cells were treated with ethanol extracts of YXT and its selected components for 7 days. Cell lysates were subjected to Western blotting with the indicated antibodies. β-actin served as a loading control. Quantifications of immunoreactive band intensities were normalized against β actin and shown in the panels: **A** NF-L, **B** NF-M, **C** NF-H, **D** β-(III)tubulin, **E** GFAP; values represent fold-change as compared to basal (Veh) in the absence of herbal treatment. *P < 0.05 (ANOVA); n = 3

Neurodegeneration is a critical pathological change in AD, and the progressive loss of structures and function in neurons is one of the main drivers of cognitive impairment in AD patients. To counter this pathological process, one approach is to promote neural differentiation. If YXT and its herbal components can promote neural differentiation, the levels of neuronal markers are expected to increase in RDIN cells that are induced to differentiate for 7 days in the presence of the herbal extracts. Western blotting analyses of lineage markers were thus performed on the RDIN culture (Fig. 3); β(III)-tubulin was used as a marker of immature neurons while various forms of neurofilaments (NF-L, -M, and -H) represent endogenous markers of mature neurons [25]. Quantification of the markers revealed that ethanol extracts of YXT and RP significantly promoted the expression of neurofilaments by about threefold and sevenfold, respectively (Fig. 3A–C). No upregulation of β(III)-tubulin was detected in YXT- and RP-treated groups (Fig. 3D). Other herbal extracts (PA, PS, RA, and RC) induced an increase in β(III)-tubulin expression by 1.7- to 2.5-fold (Fig. 3D), while no observable changes in the relative amounts of NF-L, -M, and -H were observed (Fig. 3A–C). Glial fibrillary acidic protein (GFAP) is a major type II intermediate filament found in astrocytes, and it serves as a marker of astrocytes. The amount of GFAP in the RP-treated group was reduced, but the response was not statistically significant, while the levels of GFAP in other herbal treatment groups were similar to the basal (Fig. 3E). These results suggested that PA, PS, RA, and RC increased the number of immature neurons, but not mature neurons nor astrocytes. The significant increase of NFs, as a result of RP treatment, implied that RP increased the number of mature neurons in RDIN culture. This is in line with our observation that 7 day RP treatment enhanced expression of ChAT, an endogenous marker of cholinergic neurons (which is a type of mature neuron; Fig. 2D). Although increase in NFs were also observed in the YXT-treated group (Fig. 3A–C), YXT did not alter the level of ChAT after 7 day herbal treatment (Fig. 2D), thus the YXT-induced mature neuron might not be cholinergic.

### YXT and its herbal components ameliorate NMDA-induced neuronal cell death

**Fig. 4:**
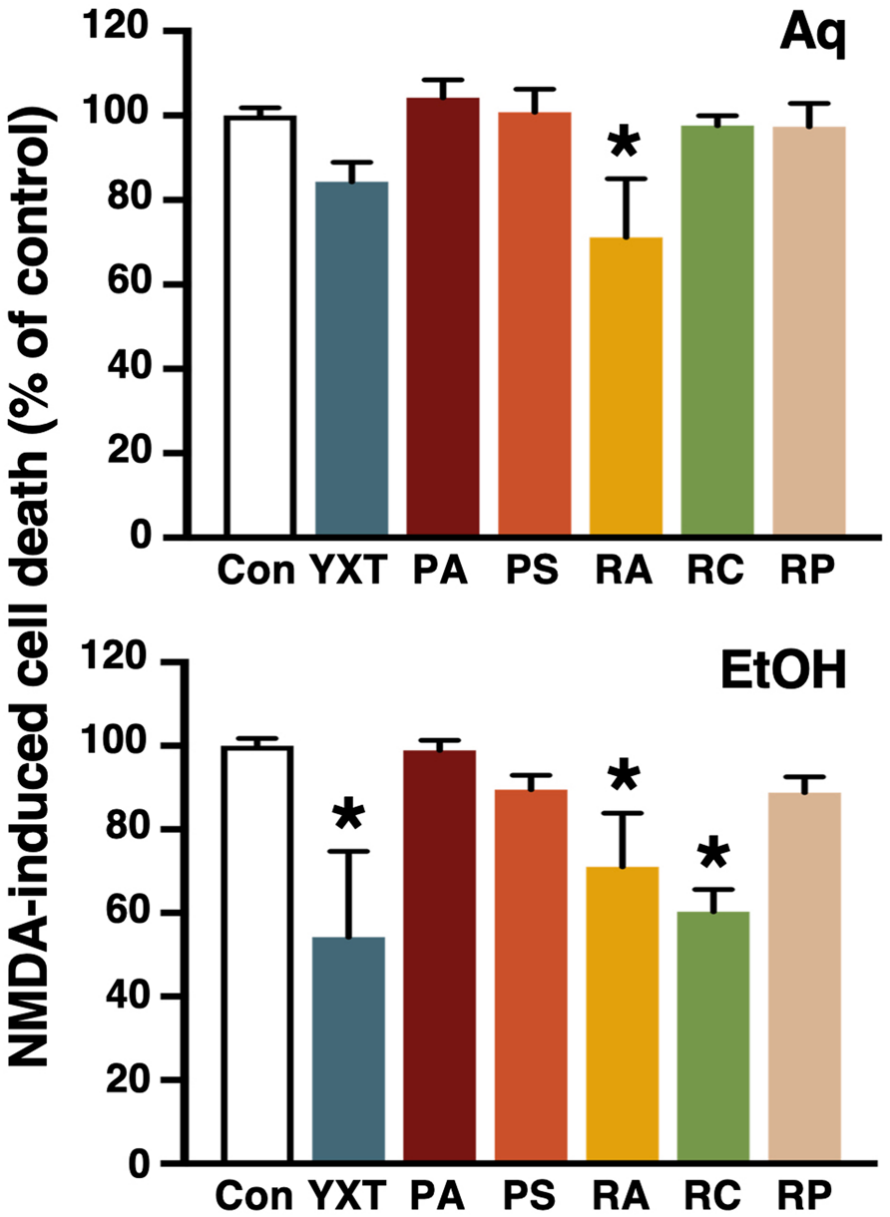
Effects of YXT and its selected herbal components on NMDA-induced primary neuronal cell death. To monitor the level of cell death induced by 100 μM NMDA (48 h), a WST assay was used to measure cell viability of the NMDA-treated primary neuronal cultures in the absence or presence of aqueous (upper; Aq) and ethanol (lower; EtOH) extracts of YXT and its selected herbal components. NMDA-induced cell death observed in the absence of herbal treatment was defined as 100%. *P < 0.05 (ANOVA); n = 6

NMDA receptor (NMDAR) is one of the major ionotropic receptors activated by glutamate and it has a fundamental role in synaptic plasticity, learning and memory. Over-activation of NMDAR results in a cascade of cellular events including apoptosis and cellular toxicity, which are commonly observed in neurodegenerative diseases including AD [26]. To elucidate the effects of YXT and its selected herbal components on NMDA-induced cytotoxicity, a WST-1 cell viability assay was performed to monitor the level of cell death induced by 100 μM of NMDA with or without a 48-h herbal treatment (Fig. 4). Significant improvement of cell viability by the aqueous extract of RA, but not other aqueous herbal extracts, was observed (Fig. 4, upper). In contrast, ethanol extracts of YXT, RA and RC significantly attenuated NMDA-induced cell death (Fig. 4, lower).

### YXT and its herbal components inhibit COX-1 and COX-2 activities

**Fig. 5:**
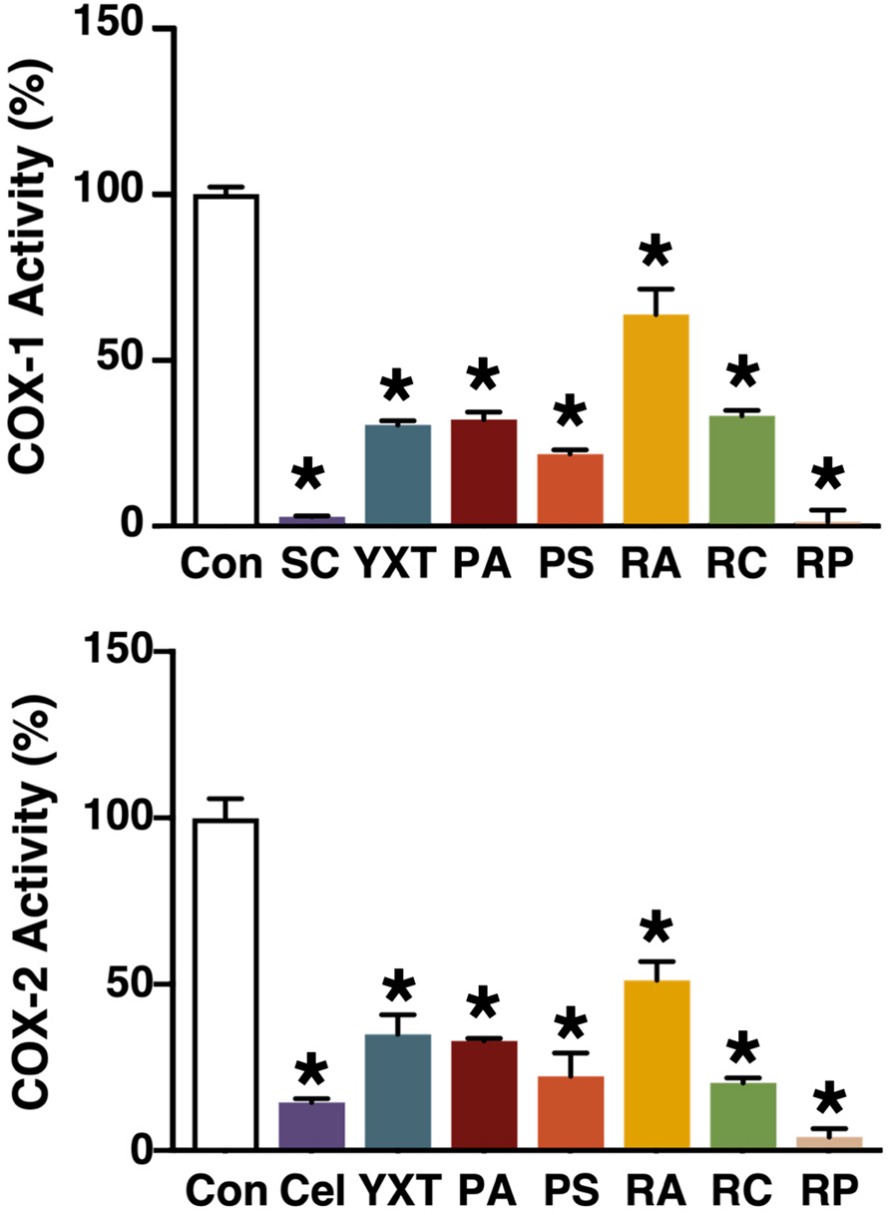
Effects of YXT and its components on COX-1 and COX-2 activities. COX-1 and COX-2 activity assay kits were used to monitor the COX enzymatic activities. Ethanol extracts of YXT or its herbal components (100 µg/mL) were mixed with respective COX enzymes (COX-1 or COX-2), COX assay buffer, arachidonic acid in NaOH, and a fluorescence probe. Fluorometric reading of enzyme control (Con) was defined as 100% COX activity. COX-1 specific inhibitor SC560 (SC) and COX-2 specific inhibitor celecoxib (Cel) served as controls. *P < 0.05 (ANOVA); n = 7

Accumulating evidence suggests the two COX enzymes (COX-1 and COX-2) are involved in the pathophysiology of neuroinflammation. Although the predominance of COX-1 or COX-2 in neuroinflammatory processes remains controversial, several studies have demonstrated that both COX-1 and COX-2 inhibitors can ameliorate cognitive decline via reducing neurotoxic Aβ generation [27] and pro-inflammatory cytokines [28]. Using a COX assay kit, the ability of YXT and its herbal components in suppressing COX-1 and COX-2 were assessed based on the fluorometric detection of prostaglandin G2, the intermediate product generated by COX enzymes. The ethanol extracts of YXT and its herbal components significantly reduced the COX-1 and COX-2 activities (Fig. 5). RP caused the most significant reduction in COX enzymatic activities, which is comparable to the known COX-1 and COX-2 inhibitors, SC560 and celecoxib. The aqueous extract of RP conferred a 60–70% inhibition on COX activities, while other aqueous extracts showed no significant inhibition. Additionally, we obtained preliminary data suggesting this inhibitory action was dose-dependent (data not shown).

### YXT and its herbal components exert multi-target effects on gene regulatory elements related to AD progression

To further elucidate the mechanisms of action of YXT and its herbal components, we examined the abilities of the herbal extracts to modulate the activities of nine gene regulatory elements that are known to (1) stimulate neurogenesis, (2) regulate neuronal cell loss, or (3) inhibit the formation of Aβ plaques and neurofibrillary tangles. We employed a well-established cellular model system, the HEK293 cell, to examine the abilities of these regulatory elements to drive the expression of luciferase. HEK293 cells stably expressing the different reporter genes were cultured in the absence or presence of herbal extracts for 24 h and then assayed for luciferase activity.

The activities of activator protein 1 response element (AP-1-RE), cAMP-responsive element (CRE), and signal transducer and activator of transcription 3 response element (STAT3-RE) were chosen as surrogate indicators of neurogenesis. AP-1 is a transcription factor that regulates differentiation and proliferation, while CRE is known to drive the transcription of brain-derived neurotrophic factor [29], which stimulates neurogenesis. Activities of these two gene regulatory elements were expected to be stimulated by anti-AD agents. The ethanol extract of YXT significantly stimulated AP-1-RE and CRE activities (Table 1). We then asked which of the selected individual herbs contributed to this activity of YXT. The AP-1-RE activity was significantly elevated by aqueous extracts of PA, and by the ethanol extracts of RA and RP (Table 2). Additionally, ethanol extracts of PA, RA and RP significantly stimulated the transcriptional activity of CRE (Table 2). On the contrary, STAT3 is known to mediate astrogliosis and its inhibition was found to ameliorate cognitive impairments in AD mouse models [30] and promote neurogenesis in neural stem cells [31]. Thus, repression of STAT3-RE expression by YXT may confer anti-AD activity. However, the transcriptional activities of the STAT3-RE were not affected by the herbal extracts except for a small reduction of activity (~ 16%) by the aqueous extract of RP (Table 2).

**Table 1:** Effects of YXT on the luciferase-linked promoter activities of gene regulatory elements in HEK293 cells

Promoter	% Luciferase activities changes^a,b^			
	Aqueous extract		Ethanol extract	
	YXT	Positive control	YXT	Positive control
Neurogenesis				
AP-1-RE	− 5.6 ± 0.1	66.3 ± 2.1^c^	36.8 ± 4.2^c^	118.7 ± 0.5^c^
CRE	− 1.7 ± 2.6	96.9 ± 2.6^c^	15.6 ± 5.0^c^	153.2 ± 0.1^c^
STAT3-RE	− 8.3 ± 1.9	56.7 ± 5.3^c^	− 11.8 ± 1.2	326.7 ± 2.9^c^
Neuronal cell survival				
LILRE	6.4 ± 1.4	30.8 ± 2.5^c^	− 3.1 ± 0.1	211.4 ± 2.7^c^
p53-RE	− 6.9 ± 1.4	166.9 ± 5.2^c^	− 49.1 ± 0.1^#^	101.5 ± 7.8^c^
TARE	− 11.7 ± 0.4	3612.2 ± 13.6^c^	− 23.2 ± 1.1^#^	2961.8 ± 57.6^c^
Aβ and NFT formation				
ISRE	− 4.2 ± 0.1	2504.0 ± 47.2^c^	4.1 ± 8.0	2753.9 ± 431.6^c^
GAS	3.4 ± 2.5	29.8 ± 7.1^c^	− 17.1 ± 5.0^d^	18.6 ± 4.3^c^
NFκB-RE	− 14.4 ± 1.8^d^	187.2 ± 8.5^c^	− 11.0 ± 0.5	149.8 ± 2.5^c^

^a^Change in activity as compared to the corresponding basal. Data represent the mean ± S.E. of quadruplicate determinations in independent experiments (n = 4)

^b^Basal activity (RFU): AP-1-RE and CRE: 10,500–30,000; LILRE, p53-RE, and TARE: 1000–5000; ISRE, and GAS and NFκB-RE: 100–500

^c^Significantly higher than the basal value (ANOVA and Dunnett’s test, *P* < 0.05)

^d^Significantly lower than the basal value (ANOVA and Dunnett’s test, *P* < 0.05)

**Table 2:** Effects of the selected YXT herbal components on the activities of gene regulatory elements

Promoter	% Luciferase activity changes ^a,b^					
		PA	PS	RA	RC	RP
Neurogenesis						
AP-1-RE	Aq	15.4 ± 0.4^c^	5.6 ± 1.0	− 1.0 ± 1.3	1.0 ± 0.4	− 6.2 ± 1.7
	EtOH	− 6.4 ± 0.5	− 10.7 ± 0.4	34.2 ± 5.6^c^	− 11.9 ± 1.8	36.8 ± 0.2^c^
CRE	Aq	− 3.5 ± 2.0	− 4.3 ± 1.9	− 6.5 ± 2.5	− 9.8 ± 1.1	− 0.9 ± 3.0
	EtOH	20.9 ± 1.8^c^	0.8 ± 0.7	17.2 ± 2.8^c^	9.0 ± 2.3	34.8 ± 0.3^c^
STAT3-RE	Aq	4.7 ± 5.2	− 12.2 ± 1.8	0.5 ± 1.8	− 6.1 ± 3.9	− 16.4 ± 3.2^d^
	EtOH	− 2.2 ± 1.7	− 11.3 ± 1.1	12.7 ± 1.2	5.9 ± 2.7	0.6 ± 1.6
Neuronal cell survival						
LILRE	Aq	11.1 ± 0.5	4.8 ± 3.7	10.2 ± 1.0	7.8 ± 2.5	3.3 ± 0.2
	EtOH	− 23.6 ± 0.6^d^	13.9 ± 3.4	2.0 ± 0.7	13.3 ± 2.7	0.4 ± 0.5
p53-RE	Aq	7.7 ± 0.4	− 5.4 ± 3.0	2.3 ± 0.9	7.1 ± 0.9	1.9 ± 2.4
	EtOH	− 55.5 ± 0.9^d^	− 31.1 ± 0.4^d^	− 11.3 ± 2.1	− 24.0 ± 1.3^d^	− 41.7 ± 1.8^d^
TARE	Aq	− 21.7 ± 1.3	− 24.0 ± 3.8	− 3.2 ± 0.7	− 7.5 ± 0.4	− 2.3 ± 1.0
	EtOH	− 14.1 ± 3.2	− 3.8 ± 6.8	− 25.3 ± 0.2^d^	− 8.4 ± 2.3	− 36.5 ± 1.7^d^
Aβ and NFT formation						
ISRE	Aq	19.7 ± 5.1	43.3 ± 16.6^c^	34.9 ± 11.7^c^	8.6 ± 4.6	69.4 ± 1.0^c^
	EtOH	2.9 ± 1.6	− 16.0 ± 3.3^d^	− 5.0 ± 9.8	4.2 ± 2.1	30.3 ± 2.6^c^
GAS	Aq	− 3.6 ± 0.5	8.2 ± 6.5	0.3 ± 2.2	− 3.3 ± 3.8	14.4 ± 12.1
	EtOH	− 16.9 ± 1.0^d^	− 23.2 ± 1.7^d^	2.5 ± 4.9	− 18.4 ± 2.0^d^	− 3.6 ± 1.1
NFκB-RE	Aq	− 13.8 ± 1.2^d^	− 17.7 ± 1.2^d^	− 4.2 ± 2.3	− 8.3 ± 2.4	− 14.3 ± 1.5^d^
	EtOH	− 22.7 ± 1.4^d^	− 6.6 ± 3.5	21.6 ± 3.6^c^	− 7.6 ± 5.0	− 16.5 ± 0.9^d^

Changes in luciferase activities induced by aqueous (Aq) and ethanol (EtOH) extracts are shown. Data are presented and analyzed as in Table 1 (n = 4)

^a,b,c,d^Please refer to Table 1 for details

Neurodegeneration is a pathological hallmark of AD with multiple causative mechanisms that involve various response elements including LPS/IL-1 response element (LILRE), TGF-β/Activin/SMAD2/SMAD3/SMAD4 response element (TARE), and p53 response element (p53-RE) Suppression of interleukin-1β (which stimulates LILRE) or p53-RE is expected to reduce apoptotic cell death [32, 33]. A decline of p53-RE activity by 49% was indeed observed in the YXT-treated group (Table 1), while selected ethanol herbal extracts, other than RA, also led to a reduction of p53-RE activity (Table 2), indicating that apoptotic events might be suppressed by these herbal extracts in HEK293 cells. Most herbal extracts did not affect the transcriptional activity of LILRE, albeit ethanol extract of PA significantly inhibit LILRE activity (Table 2). Depending on the cell type, TARE activity has been reported to promote apoptosis [34] as well as to confer a neuroprotective effect [35]. The aqueous herbal extracts did not trigger significant change in the TARE activity, whereas significant inhibitions were observed in RA and RP ethanol extracts-treated groups (Tables 1, 2).

Since hyperphosphorylation of tau protein can be stimulated by viral infections [36], activation of the interferon-stimulated response element (ISRE) and γ-activated response element (GAS) may reflect potential anti-AD actions. ISRE was stimulated by the aqueous extract of PS, RA and both extracts of RP, and inhibited by ethanol extracts of PS (Tables 1, 2). However, GAS was suppressed by the ethanol extracts of YXT, PA, PS, and RC (Tables 1, 2). The demonstrated involvement of the inflammatory response in the formation of Aβ plaques [37] suggests that suppression of pro-inflammatory nuclear factor κB response element (NFκB-RE) may alleviate AD symptoms. NFκB-RE activities were indeed suppressed by the aqueous extract of YXT and its herbal components (except RA and RC) (Tables 1, 2). As for the ethanol extract treated groups, PA and RP triggered a significant decrease in the NFκB-RE activity, but the RA-treated group showed an increase in the transcriptional activity of NFκB-RE.

### YXT and its herbal components stimulate CREB and ERK phosphorylation

**Fig. 6:**
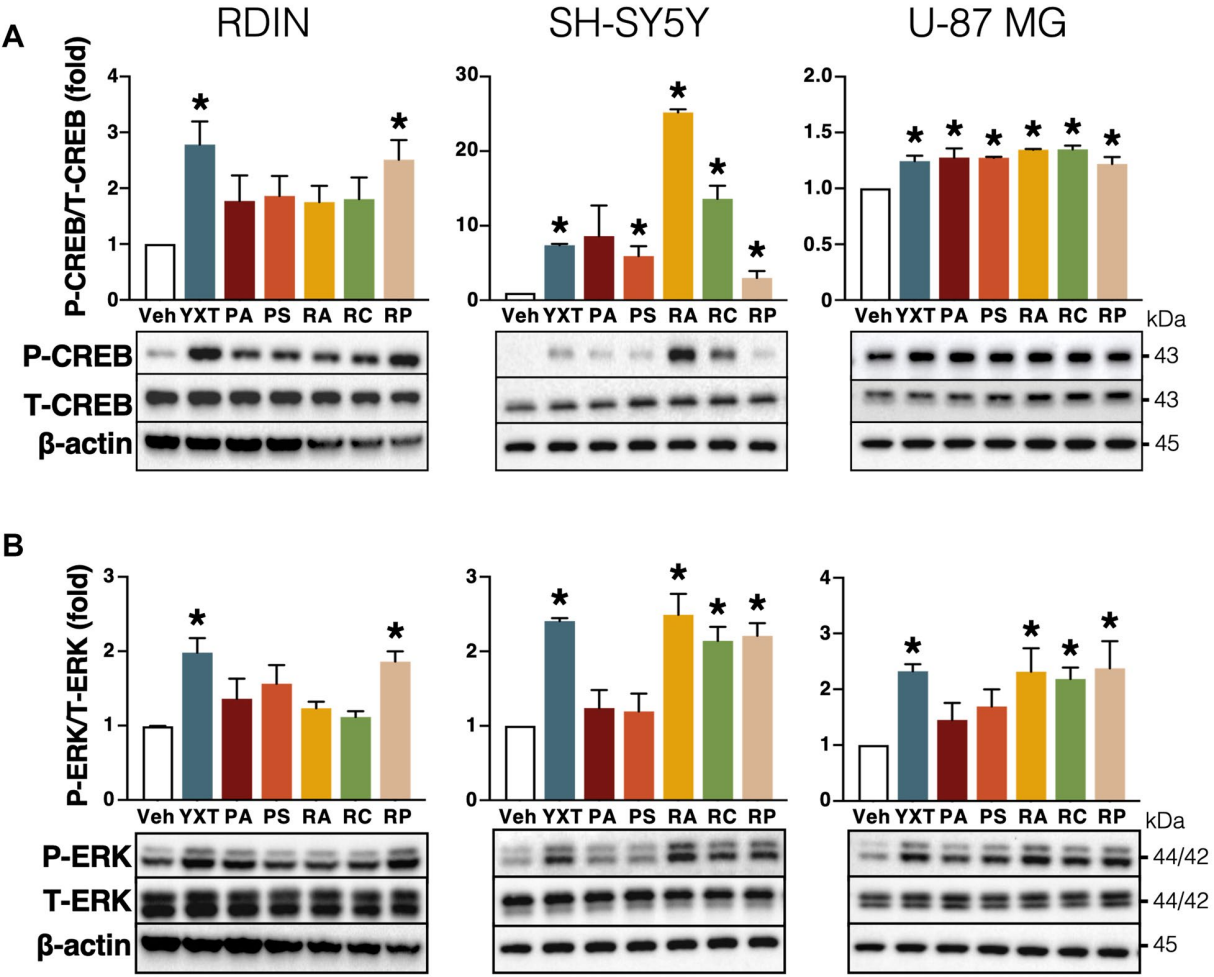
Effects of YXT and its selected herbal components on CREB and ERK phosphorylation. RDIN culture, SH-SY5Y and U-87 MG cell lines were treated with ethanol herbal extracts for 10 min. Western blotting analysis of (**A**) P-CREB and (**B**) P-ERK are shown. β-actin served as a loading control and the amount of phosphoprotein was normalized against total amount of the respective protein as a measure of the phosphorylation activity. Bar graphs illustrate the relative fold change of band intensities detected by anti-phosphoantibodies as compared to the basal group (Veh) detected in the absence of herbal treatment. *P < 0.05 (ANOVA); n = 3

Given that YXT and its constituent herbs promote the transcriptional activity of CRE (Table 2), we examined whether these herbal extracts would promote the activation of CREB, the nuclear transcription factor that binds to CRE, in RDIN culture, SH-SY5Y and U-87 MG cell lines. The levels of phosphorylated CREB were significantly promoted in all YXT- and RP-treated groups (Fig. 6A), albeit the effect of other YXT herbal components varied among cell lines and culture. In RDIN, YXT herbal constituents including PA, PS, RA, and RC did not change the phosphorylation of CREB. However, PS, RA, and RC significantly increased the amount of phospho-CREB in both SH-SY5Y and U-87 MG cells, while PA only significantly activated CREB in U-87 MG.

Since extracellular signal-regulated kinases (ERK1/2) play a major role in neuronal survival and differentiation [38], we also examined the effects of herbal extracts on ERK 1/2 phosphorylation. YXT and RP treatments significantly enhanced the phosphorylation of ERK (Fig. 6B). Similar to CREB, activation of ERK also differs among RDIN, SH-SY5Y and U-87 MG cell lines. RA and RC significantly enhanced the phosphorylation of ERK in both SH-SY5Y and U-87 MG cell lines, but not in RDIN. As for PA- and PS-treated groups, no significant change in ERK phosphorylation was observed with all cell lines and culture.

## Discussion

**Fig. 7:**
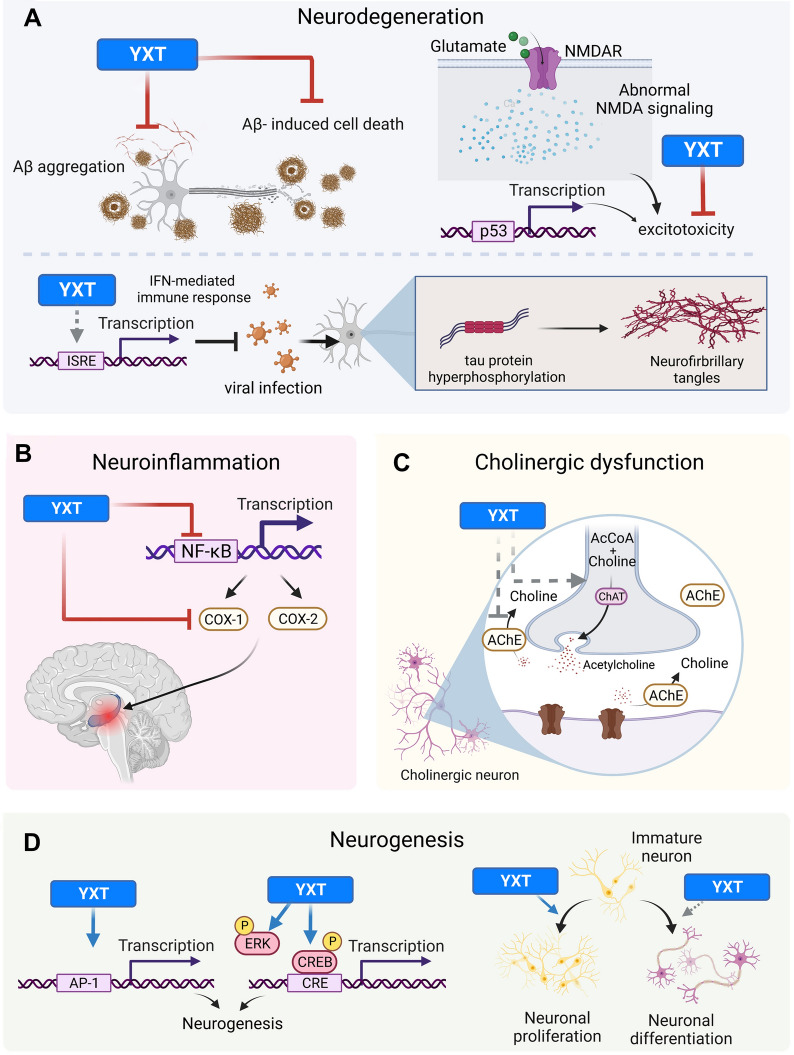
Schematic representation of the neuro-pharmacological effects of YXT on multiple molecular targets related to AD. The anti-AD effect of YXT on regulating (**A**) neurodegeneration, (**B**) neuroinflammation, (**C**) cholinergic dysfunction, and (**D**) neurogenesis. Activations are indicated by arrows, and blunt-end arrows represent inhibition. Potential activities of YXT as suggested by the actions of its component herbs are shown as dotted lines. The illustration was created with BioRender.com

YXT is a composite formula designed for treating neurasthenia that are caused by deficiency of Qi and blood of the heart. Although YXT has been used clinically for more than 700 years, the effects of YXT and its herbal components on cellular functions and signaling pathways are rarely studied. Through a panel of biochemical and cellular assays, we have demonstrated that YXT possesses anti-AD activities (Fig. 7), with several of its constituent herbs exhibiting regulatory actions on multiple targets that are implicated in AD pathogenesis. YXT and its herbal components were shown to (i) inhibit Aβ aggregation and cytotoxicity; (ii) suppress AChE activity and induce ChAT expression; (iii) exert neuroprotective effects against NMDA-induced cytotoxicity; (iv) inhibit the activities of pro-inflammatory COX-1 and COX-2 enzymes; (v) promote the differentiation of immature neurons into mature neurons; (vi) activate the neuroprotective ERK and CREB pathways; and (vii) regulate transcriptional activity of multiple intracellular targets. Collectively, these observations support the notion that YXT has beneficial actions against AD.

Ample evidence suggests that Aβ plays a central role in AD progression, with neuronal injury caused by Aβ oligomerization being one of the primary causes of neurodegeneration in AD [39]. Our demonstration that ethanol extracts of YXT, PS and RP inhibited the aggregation of Aβ (Fig. 1B) is in line with previous literature [40]; for example, a pectin extracted from RP has been shown to block the aggregation of Aβ42 via enhancing insulin-degradation enzyme and neprilysin [41]. Moreover, the ethanol extracts of YXT, PA, and PS were shown to confer a neuroprotective effect against the Aβ-induced cytotoxicity (Fig. 1B). The aqueous extract of PA is known to protect PC12 cells from Aβ42-induced cytotoxicity [42], but this was not observed in our primary neuronal cultures (Fig. 1A); this discrepancy might be attributed to the use of different cellular models as immortalized cells are typically more robust than primary cells. It is noteworthy that RP inhibited the Aβ aggregation but did not suppress the cytotoxicity caused by Aβ in the cellular assays, and differences in efficacies of the aqueous and ethanol extracts were observed. To comprehensively evaluate the anti-AD effects of YXT and its herbal components, two herbal extract preparation methods were used in this study. Aqueous extraction was employed as it is the most common method to prepare TCM decoctions for oral administration, while ethanol can maximize the extraction of hydrophobic constituents [43]. Ethanol extracts generally confer more potent anti-AD activity than aqueous extracts of the same herb, implying that the active components are relatively hydrophobic. However, an exception was observed in the case of RP, where the aqueous extract was more effective than the ethanolic extract in suppressing AChE activity (Fig. 2A). With respect to Aβ aggregation and neurotoxicity, our findings suggest that the ethanolic, instead of aqueous extracts, of YXT and its herbal components PA and PS can suppress the formation of neurotoxic Aβ aggregates and reduce the subsequent loss of neurons (Fig. 7A, upper left).

Besides the neurotoxic Aβ, dysfunctional NMDA receptor (NMDAR) signaling also results in neurodegeneration and cognitive impairment [26]. Protection against NMDA-induced toxicity was provided by the ethanol extract of YXT (Fig. 4), which might be attributed to RA and RC, since their ethanol extracts exerted similar protective effects against NMDA (Fig. 4). Formononetin, a water-insoluble isoflavone found in RA, is known to possess neuroprotective actions against NMDA [44]. Since the aqueous extract of RA was as effective as the ethanol extract (Fig. 4), it implied formononetin might not be the only bioactive that contributes to the protective effect of RA, but other hydrophobic bioactives in RA might also confer protection against NMDA. The precise mechanism by which YXT (as well as RA and RC) modulates NMDAR signaling requires further investigation. Interestingly, interleukin-1β is known to increase the NMDAR-mediated intracellular calcium [45] and activates p53, eventually leading to excitotoxic neurodegeneration [32]. Hence, suppression of p53 activation and interleukin-1β-mediated gene transcription might confer protection against NMDA. In accordance with this postulation, the ethanol extract of YXT significantly inhibited the transcriptional activity of p53-RE (Table 1), similar significant inhibitions were also observed in the PA-, PS-, and RP-ethanol extract treated groups (Table 2). Unlike most of the herbal extracts tested, the ethanol extract of PA significantly inhibited LILRE (Table 2). However, it should also be noted that aqueous extracts of PA and RA, as well as the ethanol extract of PS, weakly but significantly stimulated the LILRE activity (Table 2), while YXT did not promote the transcriptional activity of LILRE (Table 1). Taken together, these findings raise the possibility that YXT and its herbal components might confer protection against NMDA-induced neuronal excitotoxicity via complex signaling networks involving interleukin-1β and p53 (Fig. 7A, upper right).

Intracellular formation of NFT in cerebral cortex is another common cause of neurodegeneration. The formation of NFT is largely a consequence of the aggregation of hyperphosphorylated tau proteins. Recent studies have demonstrated that viral infection (e.g., HSV-1) is one of the possible causes and risk factors of the tau hyperphosphorylation [36]. ISRE and GAS are response elements for type I and type II interferons (IFNs) which are known to exert protective effects against viral infections by stimulating the immune response. Activation of ISRE and suppression of GAS transcriptional activities by YXT and its herbal components (Tables 1, 2) indicate specific effects on type I, rather than type II, IFN response. Transcription of GAS is activated by the IFN-γ-mediated JAK/STAT pathway, thus the lack of stimulation in GAS transcriptional activity implied that IFN-γ might not participate in the actions of YXT and its individual herbs. Instead, production of type I IFN and the subsequent activation of its downstream signaling pathways (e.g., PI3K pathway) [46] might be stimulated by the herbal treatments. YXT component herbs seemingly possess anti-viral activity via increased production of type I IFN, which may help to reduce the propensity of viral-induced tau hyperphosphorylation (Fig. 7A, bottom).

Association of neuroinflammation with the progression of AD is being increasingly appreciated [47]. Induction of pro-inflammatory genes mediated via the NFκB-RE has been shown to trigger neuroinflammation and deposition of Aβ [48], and at least 11 anti-AD drugs are capable of inhibiting the NFκB signaling pathway [49]. The suppression of NFκB-RE by aqueous extracts of PA, PS, and RP (Table 2) suggested that these three herbs (as well as YXT; Table 1) may possess anti-inflammatory actions (Fig. 7B). The aqueous extract of PA has a proven ability to inhibit the NFκB pathway [50, 51], but the identity of the bioactive molecule remains elusive. Tenuigenin, a constituent of RP, has been shown to attenuate microglia activation via suppressing NLR family pyrin domain-containing protein 3 inflammasome [52]. Interestingly, tenuigenin is known to inhibit the NFκB pathway [53]. Additional studies are required to verify if the action of tenuigenin is directed at the NFκB-RE. Although RC contains senkyunolide A and Z-ligustilide (two phthalides known to inhibit the production of pro-inflammatory mediators in microglia [54]), its aqueous and ethanol extracts failed to inhibit the activity of NFκB-RE (Table 2). Given that the study on microglia used purified senkyunolide A and Z-ligustilide at 50 μg/mL [54], it is reasonable for RC extracts to be ineffective at 100 μg/mL when individual phthalides are estimated to be no more than 1.5% by weight in the herb. COX enzymes are major pro-inflammatory targets downstream of NFκB. COX-1 expressing microglia are found to surround Aβ plaques [55], while COX-2 is over-expressed in activated microglia and neuron during neuroinflammation [56]. Inhibition of COX might help to impede AD progression by limiting the extent of neuroinflammation. The outstanding abilities of YXT and its herbal components to suppress COX-1 and COX-2 activities (Fig. 5) are consistent with their inhibitory action on the NFκB-RE.

The cholinergic system dysfunction greatly affects the severity of cognitive impairments in AD patients. The cholinergic hypothesis suggests that reduction in choline uptake, acetylcholine release and loss of cholinergic perikarya are the main cholinergic deficits that lead to AD progression [57]. Such deficiencies in cholinergic neurotransmission may be compensated by YXT through inhibition of AChE or enhancement of ChAT expression in cholinergic neurons (Fig. 7C). Numerous TCM-derived alkaloids have been reported to inhibit AChE, and synergistic effects have been observed between some of them [58]. Although YXT did not suppress the AChE activity, PA, PS, and RP significantly inhibited the enzyme’s activity (Fig. 2A). This might be explained by the reduced percentage by weight of individual herbs in the YXT extracts as compared to the single herb extract, resulting in insufficient amounts of the active molecule(s) to produce a detectable inhibitory effect on AChE. Additionally, it has been reported that three chemical constituents of PA, dehydroeburicoic acid, 6α-hydroxypolyporenic acid C, and pachymic acid stimulate the activity of nicotinic acetylcholine receptor [59]. Thus, PA may have a dual effect on regulating the cholinergic pathway. In our study, RP induced ChAT expression in RDIN cells upon 7 days of treatment (Fig. 2D). This might reflect an enhanced differentiation of immature neurons into cholinergic neurons, or simply an increased expression of ChAT in the differentiated neurons. Our finding is supported by a recent report which showed that the RP ethanol extract can significantly increase ChAT level and acetylcholine production in the prefrontal cortex of scopolamine-treated mice [60]. Taken together, the involvement of RP in enhancing cholinergic neurotransmission seemed credible.

Diminished neurogenesis in adult hippocampus is considered as an early event of AD pathogenesis [61], and thus stimulation of neuronal differentiation and/or neurogenesis may slow down disease progression. The differentiation of neural stem cells and progenitor cells will result in an increase in the number of new mature neurons. The RP-induced expressions of neurofilaments (Fig. 3A–C) are in line with previous findings [16, 62]. Two bioactives extracted from RP, namely polygalasaponin G [63] and tenuigenin [62], are candidate compounds for these effects. As proliferation of neural progenitor cells is reflected by an increase in β(III)tubulin expression, ethanol extracts of PA, PS, RA, and RC appeared to stimulate neurogenesis (Fig. 3D); astrocytic lineage was not increased since the amount of GFAP was unaltered by the extracts (Fig. 3E). According to Song et al. [64], the β(III)-tubulin expression of RDIN remains unchanged in the late differentiation stage (7–15 days after withdrawal of growth factors). This suggests that the observed changes are induced by the herbal treatment, instead of the normal differentiation of RDIN cultures. Kenpaullone, which is known to increase the number of proliferative cells in ReNcell-derived cultures [65], is reported to have similar effects on β(III)tubulin and GFAP levels. However, neuronal maturation was unlikely to be promoted by these herbal extracts because no increase in neurofilaments was detected (Fig. 3A–C). Astragaloside VI, a bioactive isolated from RA, is found to stimulate the proliferation of neural stem cells [66]. This is consistent with our observation on the increase of immature neurons induced by RA. Collectively, the enhanced differentiation to mature neurons elicited by YXT and its herbal components warrants further investigations on their neurogenic actions. Besides differentiation into cholinergic neurons, the effects of herbal extracts on other types of mature neurons, including dopaminergic, GABAergic, glutamatergic, and serotonergic neurons, should be examined. These studies may provide additional insights on how YXT and its herbal components may promote neuronal differentiation and reduce neuronal loss (Fig. 7D, right).

Activation of neurotrophin response represents a therapeutic approach to alleviate the effect of diminished neurogenesis on cognitive impairment in AD patients. CRE is activated by the binding of phosphorylated CREB, which is a major mediator of neurotrophin responses [67]. In vivo studies have revealed a reduction of CREB activation in AD mouse models [68]. Our luciferase assays indicated that the ethanol extracts of YXT, PA, RA and RP significantly promoted the transcriptional activity of CRE (Tables 1, 2). This is further supported by our observation that YXT and RP stimulated the phosphorylation of CREB in RDIN, SH-SY5Y, and U-87 MG cells (Fig. 6A). Activation of the CRE/CREB transcriptional pathway is known to exert neuroprotection against oxidative stress-mediated neuronal cell death [69], and it also plays a direct role in the memory-related synaptic plasticity [70]. The enhanced CRE and CREB activity induced by YXT and its herbal components (PS, RA, RC and RP) may contribute to their neurogenic effects (Fig. 7D, left). We have previously shown that astragaloside IV from RA can activate the phosphorylation of CREB in various cell types including primary neurons [71]. Two components of RP (3,6’-disinapoyl sucrose and tenuifoliside A) have previously been shown to stimulate the phosphorylation of CREB [72]. They may account for the ability of the ethanol extract of RP to stimulate CRE-driven transcription. Besides CREB, it is widely acknowledged that ERK is another key mediator of brain function and an important signaling molecule for neurotrophic responses [70]. The ability of YXT and RP to regulate ERK signaling is clearly demonstrated in RDIN, SH-SY5Y and U-87 MG cells (Fig. 6B). ERK phosphorylation induced by RP may be attributed to tenuifoliside A, an RP component known to activate ERK in C6 glioma cells [73]. Two other YXT constituent herbs (RA and RC) also stimulated ERK phosphorylation in SH-SY5Y and U-87 MG cells (Fig. 6B), and their ability to stimulate ERK phosphorylation has been previously reported [74, 75], which is in agreement with our observation. Collectively, these findings revealed that YXT and its herbal components are capable of enhancing ERK- and CRE/CREB-mediated pathways, which are beneficial in combating AD.

Among the thirteen component herbs of YXT, PA and PS are closely related. PA is the sclerotium of a woody-decay Polyporaceae fungus (Wolfiporia cocos) on pine trees, while PS (also known as Poria cum Radix Pini) is the sclerotium of the same fungus that contains part of the root of the pine tree (hostwood). Although the pharmacological properties of PA are well documented [76], PS is rarely studied. Typical usages of PA and PS are different in TCM practice. PA is used for tonifying the spleen, draining out dampness by promoting diuresis, while PS is mainly used for calming and sedative effects. The differences in PA and PS chemistry have not been fully established [77]. Although PA and PS shared a similar profile in their anti-AD activities, PS-treated groups usually showed a more significant effect than the PA (Figs. 1, 3B, 5, 6A). This implies that bioactives unique to PS, or present at higher abundance in PS, might contribute to its stronger neuroprotective effects.

This study is the first to investigate the pharmacological actions of YXT and its herbal components at the molecular level. It is worth noting that this study has several limitations. For instance, the synergistic effects of herbal components were beyond the scope of the current study. Although YXT and its herbal components showed significant multi-target anti-AD properties in vitro (Fig. 7), the in vivo anti-AD activity needs to be examined to better illustrate the therapeutic potential of YXT. To elucidate the in vivo effect of YXT, it will be desirable to evaluate its absorption, bioavailability, and distribution in rodents. Such studies will enable informed prediction of pharmacological effects and dosage determination for the testing of active extracts in animal models. Using chemical markers of the herbal components, such as 3,6-disinapoyl-sucrose polygalaxanthone III for RP and pachymic acid for PA and PS, we have obtained preliminary evidence of absorption and distribution of YXT in rodent models. The stage is set for demonstrating the effects of YXT on memory and cognition in animal behavioral models.

## Conclusions

YXT was shown to exhibit pharmacological activities at multiple molecular targets that are relevant to the pathogenesis of AD. YXT and its herbal components inhibited the aggregation and cytotoxicity of Aβ, strengthened the cholinergic signaling through suppressing AChE activity and enhancing ChAT expression, and ameliorated the cytotoxicity induced by NMDA. They also stimulated the differentiation of neural progenitor cells and inhibited COX activities. Moreover, YXT and its components altered the transcriptional activity of multiple gene regulatory elements involved in AD progression. Signaling pathways mediated by CREB and ERK were also activated by YXT and some of its herbal constituents. These findings support the potential use of YXT as an alternative medicine in treating AD and provide impetus to explore their underlying mechanism therapeutic potentials through in vivo studies in the future.

### Supplementary Information

**Additional file 1: Table S1.** Authentication of TCM herbal components by HPLC. YXT and its component herbs including PA, PS, RA, RC and RP were subjected to standard extraction, followed by HPLC analysis to detect their expected chemical markers. Chemical marker standards were used to define the retention time of each standard under the specified HPLC conditions. Results obtained on the authentication of herbal materials agreed with the corresponding HPLC profiles documented in Che et al. (2010), Duan et al. (2006), or the Chinese Pharmacopoeia (2020 edition). A C18 reverse phase column was used for the HPLC analysis. Mobile phases were applied as follows: A, acetonitrile:0.1% phosphoric acid (75:25); B, acetonitrile:water (32:68); C, methanol:acetic acid (30:70); D, methanol:0.05% phosphoric acid (70:30); E, acetonitrile:water (70:30). The flow rate was set at 0.8 or 1 mL/min as suggested in the literature. The injection volume of the samples and standard solutions was 20 μL. **Table S2.** Cytotoxic effects of herbal extracts. HEK293, SH-SY5Y, U-87 MG and RDIN cells were treated with increasing concentrations of herbal extracts (0.01 to 1,000 μg/mL) and incubated for 2 days, followed by a WST-1 cell viability assay to assess the cytotoxic effects of the herbal extracts. NS, no significant toxicity when applied up to 1 mg/mL; ND, not determined.

## Data Availability

The datasets used and/or analyzed during the current study are available from the corresponding author on reasonable request.

## Abbreviations

AD: Alzheimer’s disease
AChE: Acetylcholine esterase
Aβ: β-Amyloid
AP-1-RE: Activator protein 1 response element
CC: *Cortex Cinnamomi*
ChAT: Choline acetyltransferase
COX: Cyclooxygenase
CRE: CAMP responsive element
CREB: CAMP-responsive element binding protein
ERK: Extracellular-signal regulated kinase
FS: *Fructus Schisandrae*
GAS: γ-Activated sequence
GFAP: Glial fibrillary acidic protein
GS: *Radix Ginseng*
HEK293: Human embryonic kidney 293 cells
IFN: Interferon
ISRE: Interferon stimulated responsive element
LILRE: LPS and IL-1 responsive element
NF: Neurofilament
NFT: Neurofibrillary tangles
NFκB-RE: Nuclear factor κ-light-chain-enhancer of activated B cells response element
NMDA: N-methyl D-aspartate
PA: *Poria cocos*
PP: *Rhizoma Pinelliae Preparatum*
PS: *Poria Sclerotium pararadicis*
RA: *Radix Astragali*
RC: *Rhizoma Chuanxiong*
RDIN: ReNcell-derived immature neurons
RFU: Relative fluorescence unit
RG: *Radix et Rhizoma Glycyrrhizae*
RP: *Radix Polygalae*
SP: *Semen Platycladi*
STAT3: Signal transducer activator protein 3
SZ: *Semen Ziziphi spinosae*
TARE: TGF-β/Activin/SMAD2/SMAD3/SMAD4 response element
TCM: Traditional Chinese medicine
ThT: Thioflavin T
WST-1: Water soluble tetrazolium salts-1
YXT: Yang Xin Tang
