# Supplementary material for: Multi-target regulatory mechanism of Yang Xin Tang − a traditional Chinese medicine against dementia
Source: Chin Med. 2023 Aug 16;18:101. doi: 10.1186/s13020-023-00813-w (PMC10428601; doi:10.1186/s13020-023-00813-w)
Supplement: Supplementary file 1 — Additional file 1: Table S1. Authentication of TCM herbal components by HPLC. YXT and its component herbs including PA, PS, RA, RC and RP were subjected to standard extraction, followed by HPLC analysis to detect their expected chemical markers. Chemical marker standards were used to define the retention time of each standard under the specified HPLC conditions. Results obtained on the authentication of herbal materials agreed with the corresponding HPLC profiles documented in Che et al. (2010), Duan et al. (2006), or the Chinese Pharmacopoeia (2020 edition). A C18 reverse phase column was used for the HPLC analysis. Mobile phases were applied as follows: A, acetonitrile:0.1% phosphoric acid (75:25); B, acetonitrile:water (32:68); C, methanol:acetic acid (30:70); D, methanol:0.05% phosphoric acid (70:30); E, acetonitrile:water (70:30). The flow rate was set at 0.8 or 1 mL/min as suggested in the literature. The injection volume of the samples and standard solutions was 20 μL. Table S2. Cytotoxic effects of herbal extracts. HEK293, SH-SY5Y, U-87 MG and RDIN cells were treated with increasing concentrations of herbal extracts (0.01 to 1,000 μg/mL) and incubated for 2 days, followed by a WST-1 cell viability assay to assess the cytotoxic effects of the herbal extracts. NS, no significant toxicity when applied up to 1 mg/mL; ND, not determined. [file 13020_2023_813_MOESM1_ESM.docx]

**Table S1.** Authentication of TCM herbal components by HPLC.

| TCM | Marker | Mobile phase | Retention time (min) | Reference |
| --- | --- | --- | --- | --- |
| PA | Pachymic acid | A | 56.6 | [a] |
| PS | Pachymic acid | A | 56.6 | [a] |
| RA | Calycosin-7-glucoside | B | 11.1 | [b] |
|  | Astragaloside IV | B | 35.0 | [b] |
| RC | Tetramethylpyrazine | C | 14.7 | [b] |
|  | Ferulic acid | C | 25.1 | [b] |
|  | Z-ligustilide | C | 48.0 | [b] |
| RP | 3,6’-Disinapoyl sucrose | D | 9.3 | [b] |
|  | Polygalaxanthone III | D | 16.5 | [b] |
|  | Tenuifolin | D | 34.3 | [b] |
| YXT | Formononetin | B | 38.6 | [b] |
|  | Pachymic acid | E | 7.2 | [c] |
|  | Z- ligustilide | E | 11.6 | [c] |

YXT and its component herbs including PA, PS, RA, RC and RP were subjected to standard extraction, followed by HPLC analysis to detect their expected chemical markers. Chemical marker standards were used to define the retention time of each standard under the specified HPLC conditions. Results obtained on the authentication of herbal materials agreed with the corresponding HPLC profiles documented in Che et al. (2010), Duan et al. (2006), or the Chinese Pharmacopoeia (2020 edition). A C18 reverse phase column was used for the HPLC analysis. Mobile phases were applied as follows: A, acetonitrile:0.1% phosphoric acid (75:25); B, acetonitrile:water (32:68); C, methanol:acetic acid (30:70); D, methanol:0.05% phosphoric acid (70:30); E, acetonitrile:water (70:30). The flow rate was set at 0.8 or 1 mL/min as suggested in the literature. The injection volume of the samples and standard solutions was 20 μL.

[a] Che et al., 2010, *Acta pharmaceutica Sinica*, 45(4), 494-497

[b] Chinese Pharmacopoeia (2020 edition)

[c] Duan et al., 2006, *Chinese Traditional and Herbal Drugs*, 37(2), 284

**Table S2.** Cytotoxic effects of herbal extracts.

|  | Aqueous extract | | |  |  | Ethanol extract | | | |
| --- | --- | --- | --- | --- | --- | --- | --- | --- | --- |
| TCM | HEK293 | SH-SY5Y | U-87 MG |  | HEK293 | | SH-SY5Y | U-87 MG | RDIN |
| YXT | NS | NS | NS |  | ≥ 300 μg/mL | | ≥ 300 μg/mL | NS | ≥ 1000 μg/mL |
| PA | NS | ≥ 100 μg/mL | NS |  | ≥ 1000 μg/mL | | ≥ 300 μg/mL | ≥ 1000 μg/mL | ≥ 300 μg/mL |
| PS | NS | ≥ 100 μg/mL | NS |  | ≥ 1000 μg/mL | | ≥ 30 μg/mL | NS | ≥ 300 μg/mL |
| RA | NS | NS | NS |  | NS | | ≥ 1000 μg/mL | NS | ≥ 1000 μg/mL |
| RC | NS | NS | NS |  | ≥ 1000 μg/mL | | ≥ 30 μg/mL | ≥ 1000 μg/mL | ≥ 1000 μg/mL |
| RP | NS | ≥ 1 μg/mL | ≥ 10 μg/mL |  | ≥ 100 μg/mL | | ≥ 3 μg/mL | ≥ 30 μg/mL | ≥ 30 μg/mL |
| PP | ≥ 300 μg/mL | ND | ND |  | NS | | ND | ND | NS |
| RS | ≥ 300 μg/mL | ND | ND |  | ≥ 1000 μg/mL | | ND | ND | NS |
| RG | ≥ 1000 μg/mL | ND | ND |  | ≥ 1000 μg/mL | | ND | ND | ≥ 1000 μg/mL |

HEK293, SH-SY5Y, U-87 MG and RDIN cells were treated with increasing concentrations of herbal extracts (0.01 to 1,000 μg/mL) and incubated for 2 days, followed by a WST-1 cell viability assay to assess the cytotoxic effects of the herbal extracts.

NS; No significant toxicity when applied up to 1,000 μg/mL

ND; Not determined
